# Urinothorax: A Rare Cause of Pleural Effusion

**DOI:** 10.7759/cureus.25392

**Published:** 2022-05-27

**Authors:** Ahmad Raja, Abhinav Dhakal, Pavel Sinyagovskiy, Mohammed Abdalla, Summia Matin Afridi

**Affiliations:** 1 Internal Medicine, Bassett Healthcare Network, Cooperstown, USA; 2 Medicine, University of Arizona College of Medicine - Tucson, Tucson, USA; 3 Critical Care, Yuma Regional Medical Center, Yuma, USA; 4 Pulmonary and Critical Care, Medical College of Wisconsin, Milwaukee, USA

**Keywords:** urologic procedure, ureteral stent, obstructive hydronephrosis, rare cause of pleural effusion, urinothorax

## Abstract

Urinothorax is a rare cause of pleural effusion, which is seen in patients with obstructive uropathy, blunt trauma, or ureteric injury during abdominal surgical procedures. Clinical symptoms may include dyspnea, chest pain, cough, fever, abdominal pain, and decreased urine output. Diagnosis is made by thoracentesis, which would reveal fluid with a urine-like odor, and pleural fluid analysis, which would show if fluid is transudative in nature with a pH lower than 7.30. Pleural fluid to serum creatine ratio of more than 1 is diagnostic for this condition. In our case, the patient underwent percutaneous nephrolithotripsy with a stent placement three days before presentation to the hospital. She was diagnosed with urinothorax, which led to further investigations, and she was found to have persistent hydronephrosis. Her condition improved after her underlying hydronephrosis was addressed with stent placement. She was discharged home in stable condition.

## Introduction

Urinothorax is one of the infrequent causes of pleural effusion and is defined as the presence of urine in the pleural space. It is seen in patients with obstructive uropathy, blunt trauma, or ureteric injury during abdominal surgical procedures [1-3]. It can also occur in the setting of extra-urinary malignancies, extracorporeal shock wave lithotripsy, or after percutaneous nephrolithotomy [4-6]. Urinothorax is divided into two main groups based on the underlying cause, obstructive urinothorax (due to underlying obstructive uropathy) and traumatic urinothorax (due to injury to the urinary tract) [7,8]. Trauma to the urinary tract can cause urine leakage into the peritoneal cavity, which can accumulate in the pleural space (more commonly on the ipsilateral side) through either lymphatic connection or diaphragmatic pores [9,10].

## Case presentation

**Figure 1:**
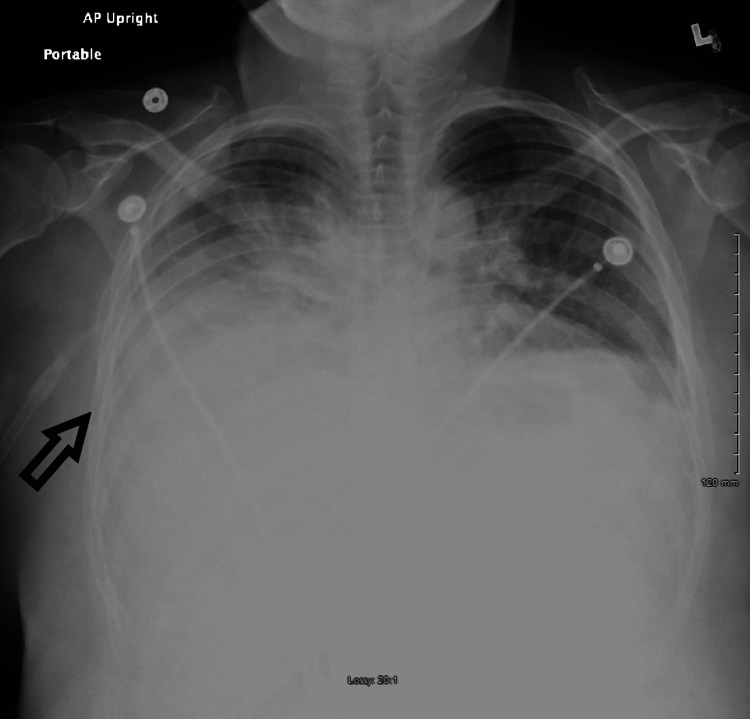
Chest X-ray showing bilateral pleural effusions with right worse than left (arrow).

**Figure 2:**
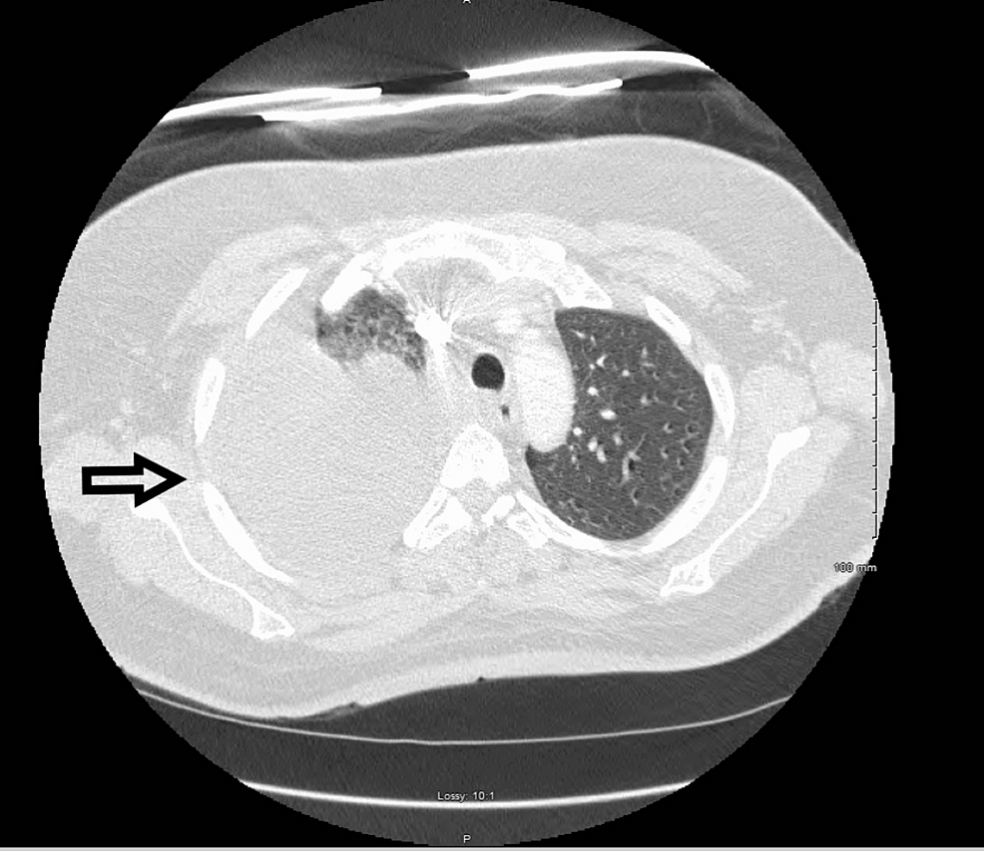
CT chest showing right-sided pleural effusion.

**Figure 3:**
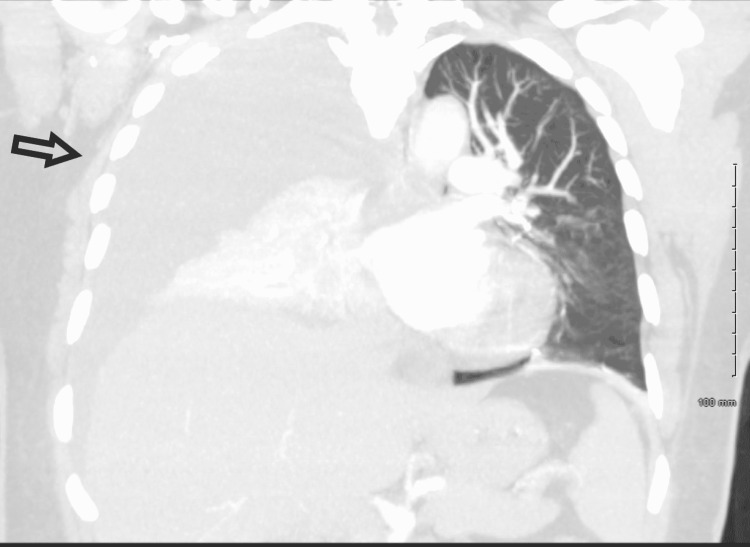
CT chest coronal view showing complete opacification and atelectasis of the right, middle and lower lobe.

A 51-year-old woman presented to the hospital with chest pain and shortness of breath for two days. She described the pain as substernal and non-radiating with 3/10 severity. There was no associated fever, chills, cough, nausea, or diaphoresis. Her history was significant for percutaneous nephrolithotripsy with a stent placement three days ago for a right-sided ureteral stone with hydronephrosis. Vitals were notable for a respiratory rate of 20 breaths per minute with a saturation of 94% on 4 liters of oxygen through the nasal cannula. Physical examination was significant for decreased breath sounds on the right lung field. Lab investigations, including complete blood and comprehensive metabolic panel, were normal. EKG was unremarkable, and troponin levels were undetectable. Chest X-ray showed bilateral pleural effusions with right worse than left (Figure 1). She was started on broad-spectrum antibiotics for presumptive diagnosis of hospital-acquired pneumonia. CT angiogram was negative for pulmonary embolism and confirmed the right-sided pleural effusion with atelectasis of the right, middle and lower lobe (Figures 2-3).

A chest tube was placed with drainage of 2500 cc of serous fluid, and analysis showed transudative fluid with a glucose of 226, protein less than 1, pH 7.23, and lactate dehydrogenase (LDH) of 511 with negative Gram stain and culture. Due to her recent urologic procedure, urinothorax was suspected as a possible etiology. Therefore, pleural fluid creatinine was checked, which was 1.3 compared to a serum creatinine level of 0.7 with a ratio of 1.8 that confirmed the diagnosis. There was persistently high output from the chest tube with 1700 cc on day one and 800 cc on day two, with minimal improvement in symptoms. Therefore, a CT urogram was done that did not show any urine leakage, but there was still persistent hydronephrosis on the right side. She was taken to the operating room (OR) for a retro pyelogram with stent placement. After the procedure, her symptoms significantly improved, and drainage from the chest tube reduced to less than 10 cc. The chest tube was removed, and she was discharged home in good condition.

## Discussion

Urinothorax is a rare cause of pleural effusion [1]. Clinical symptoms may include dyspnea, chest pain, cough, fever, abdominal pain, and decreased urine output [11]. Differential diagnosis of urinothorax includes pulmonary consolidation, hemothorax, and chylothorax [12]. Diagnosis of urinothorax is made by thoracentesis, which would reveal fluid with a urine-like odor, and pleural fluid analysis, which would show if fluid is transudative in nature with a pH lower than 7.30. Pleural fluid to serum creatine ratio of more than 1 is diagnostic for this condition [7,13]. A case of exudative fluid in urinothorax has also been reported emphasizing the point of keeping it in the differential diagnosis of exudative pleural effusion [14].

In a patient with urinothorax, diagnostic tests including renal scan, pyelogram, and contrast-enhanced CT are required to identify the location of the defect in the urinary system [5,15]. Renal ultrasound and CT abdomen can show findings consistent with hydroureteronephrosis. Contrast-enhanced CT can reveal the underlying reno-pleural fistula. Technetium-99m renal scintigraphy can demonstrate the translocation of 99Tc-labeled albumin from the genitourinary tract into the pleural space [16,17].

Management is based on correcting the underlying genitourinary tract pathology with or without drainage of the urinothorax. The prognosis depends on the underlying cause of the urinothorax. It usually resolves if it is identified and treated early before the development of pulmonary complications and if the underlying cause is promptly addressed [12].

In our case, the patient underwent percutaneous nephrolithotripsy with a stent placement three days before presentation to the hospital. She was diagnosed with urinothorax, which led to further investigation with a CT urogram and was found to have persistent hydronephrosis. However, her condition improved after her underlying hydronephrosis was addressed with stent placement.

## Conclusions

Although urinothorax is rare, it should be considered as one of the differentials in a patient presenting with pleural effusions, especially if a patient has underlying obstructive uropathy or has undergone recent abdominal surgery, as early recognition and treatment of the underlying condition can help improve outcomes.
